# The Relationship between Maternal Gestational Impaired Glucose Tolerance and Risk of Large-for-Gestational-Age Infant: A Meta-Analysis of 14 Studies

**DOI:** 10.4274/jcrpe.2583

**Published:** 2016-09-01

**Authors:** Hai-Qing Wang, Han-Lin Lai, Yi Li, Qi-Fei Liu, Shuang Hu, Li Li

**Affiliations:** 1 Anhui Medical University School of Public Health, Department of Nutrition and Food Hygiene, Anhui, China

**Keywords:** Gestational impaired glucose tolerance, large for gestational age, meta-analysis

## Abstract

**Objective:**

To explore, by conducting a meta-analysis, whether gestational impaired glucose tolerance (IGT) is an independent predictor of neonatal large for gestational age (LGA) or not.

**Methods:**

Medline, Embase, and Cochrane Library databases were searched to identify published epidemiological studies (cohort and case-control studies) investigating the association between gestational IGT and neonatal LGA. Calculations of pooled estimates were conducted in random-effect models or fixed-effects models. Heterogeneity was tested by using chi-square test and I2 statistics. Egger’s test (linear regression method) and Begg’s test (rank correlation method) were used to assess potential publication bias.

**Results:**

Fourteen observational studies were included in the meta-analysis. The overall risk for the effect of IGT on LGA was 2.09 (1.56, 2.78). Stratified analyses showed no differences regarding different geographic regions or the analysis of overall adjusted odds ratios. No evidence of publication bias was observed in either Egger’s test or Begg’s test results.

**Conclusion:**

Gestational IGT is an independent predictor of neonatal LGA.

**WHAT IS ALREADY KNOWN ON THIS TOPIC?**

Some studies demonstrated that women with gestational impaired glucose tolerance were at higher risk of adverse pregnancy outcomes while compared with women with normal glucose tolerance, but some studies supported the idea that the discrepancies of the searches may be related to the criteria used to diagnose this condition.

**WHAT THIS STUDY ADDS?**

Gestational impaired glucose tolerance is an independent predictor of neonatal large for gestational age.

## INTRODUCTION

Gestational impaired glucose tolerance (IGT) is defined as an abnormal glucose level obtained in an oral glucose tolerance test (OGTT) during pregnancy. Gestational IGT is considered to reflect a serious defect in beta-cell function in the early and late-phases of insulin secretion and is regarded as a sign of pre-gestational diabetes mellitus (GDM) (1). Universally, GDM is associated with adverse pregnancy outcomes and its incidence increased in parallel to the increase in frequency of obesity worldwide. Women identified as GDM patients are treated with dietary or insulin therapy to reduce their glucose levels and hence the risk of adverse pregnancy outcomes (2). While the importance of identification and treatment of GDM and the benefit of controlled blood glucose in the prenatal period is universally confirmed, knowledge on the mechanisms responsible for the impact of gestational IGT on pregnancy outcome is inconclusive. The offspring of women with IGT, compared to those of women who had good glucose control during pregnancy, are reported to have increased birth weights, increased rates of macrosomia, and increased frequency of large for gestational age (LGA) (3). Some studies demonstrated that women with gestational IGT were at higher risk of adverse pregnancy outcomes as compared with women with normal glucose tolerance (NGT), but others have attributed these findings to differences in the criteria used to diagnose this condition (4,5). However, there is no systematic review or meta-analysis of the studies on the importance of gestational IGT as a public health problem. With this background, we attempted to conduct a meta-analysis of the studies on the association between gestational IGT and pregnancy outcome published within the last decade.

## METHODS

We performed a detailed search on Medline, Embase, and Cochrane Library to identify articles that reported the relationships between IGT during pregnancy and neonatal outcomes. We also attempted to reach the comments on these studies through review articles. The database was searched from 1999 to April 2015 and limited to human studies which were published in English.

We used the following search terms: “gestational” or “pregnant” and “impaired glucose tolerance” or “IGT” and “large for gestational age” or “LGA”. Studies were included in the analysis if they examined outcomes in pregnant women who had IGT but not GDM and women who had not received any treatment. The primary adverse outcome searched in this meta-analysis was LGA, defined as a birth weight >90th percentile for gestational age.

Quality assessment of the available studies was conducted independently by two reviewers (Hai-Qing Wang, Han-Lin Lai) using the Newcastle-Ottawa quality assessment scale for cohort studies and for case-control studies (6). The scores range from 0 to 9 and scores ≥6 were graded as of high-quality.

**Data Extraction**

Using a standardized data-collection form, the two reviewers (Hai-Qing Wang, Han-Lin Lai) extracted the data from the searched article independently, and any disagreement was resolved by discussion. The following study characteristics were recorded: first author’s name, year of publication, country of origin, study design, inclusion and exclusion criteria, sample size, diagnostic criteria for gestational IGT, potential confounding factors adjusted for. All search results were exported to Endnote 7.0 to organized references and duplications were thus eliminated.

### Statistical Analysis

We extracted the odds ratio (OR) and the 95% confidence intervals (95% CI) to reflect the uncertainty of point estimates from each study. The crude OR for gestational IGT and LGA could be calculated from 5 studies and the other 9 studies which were stratified by some confounding factors (such as quality grade, number of confounding factors adjusted for, study population) which reported adjusted OR and the 95% CI. The chi-square test was used to analyze the heterogeneity of the results, and p<0.10 was considered as the cut-off level of heterogeneity. We also used I^2^ to judge the heterogeneity between these studies, I^2^ representing the percentage of the true heterogeneous (non-sampling error) in the total variability; when I^2^ was >50%, we recognized the existence of heterogeneity (7). When substantial heterogeneity was detected, the summary estimate on the basis of the random-effects model using the method of Der Simonian and Laird (8) was presented. These two approaches yield similar results when the heterogeneity of the study is small, the random-effects model gives more weight to imprecise (or small) studies compared to a fixed-effects model (9). In addition, the pooled estimate that was based on the fixed-effects model using the inverse variance method was presented (10). In order to assess the impact on the results of a single study, we conducted a sensitivity analysis of each study by excluding each study one by one and recalculating the combined estimates on remaining studies. We used a funnel plot (11) to visualize the publication bias and used Egger’s test (linear regression method) (12) and Begg’s test (rank correlation method) (13) to assess potential publication bias. The Egger’s test is a linear regression method about standard normal deviate and precision of all the studies in meta-analysis. The Begg’s test is a rank correlation test for inspection of the correlation of effect and sample size. When the number of the studies in the meta-analysis is <20, the effects of these two methods are low, but the sensitivity of the Egger’s test is higher than the Begg’s test. Meta-analysis was performed with Stata/SE10.0 (Stata Corp, College Station, TX, USA).

## RESULTS

**Figure 1:**
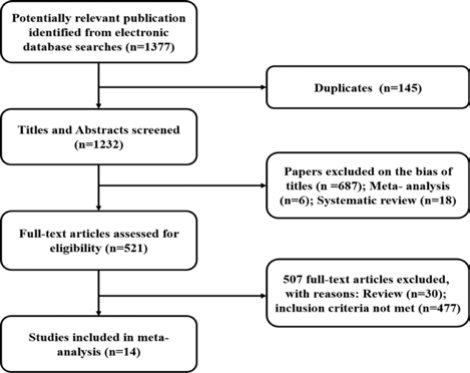
Process of literature search in our meta-analysis

In the preliminary literature search, we identified 1377 unique citations from the electronic databases (Figure 1). No supernumerary article was found in the citations by manual search and 145 were rejected because of duplicates. 711 were rejected because of 687 articles were on bias of titles, 6 studies were meta-analysis, and 18 were systematic reviews. The remaining 521 full-text articles were selected and inspected and then we excluded 507 articles because there were 30 reviews and 477 studies which did not meet the inclusion criteria of meta-analysis. Finally, we ended up with 14 observational studies (4,5,14,15,16,17,18,19,20,21,22,23,24,25) for our analysis.

The characteristics of these 14 observational studies are displayed in Table 1. There were 13 cohort studies and only one case–control study. Six of the studies were conducted in Europe, 4 in North America, and 4 in Asia. The effect of gestational IGT on LGA and the definition of gestational IGT in each study are also demonstrated in Table 1.

**Figure 2:**
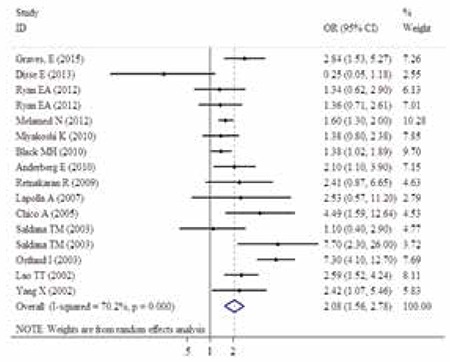
The odds ratio of large for gestational age infants in relation to gestational impaired glucose tolerance from individual studies and the overall odds ratio of these 14 observational studies

The ORs of LGA in relation to gestational IGT from each study and the overall OR are presented in Figure 2. We assembled the OR and 95% CI of the 14 studies which were related to the effect of gestational IGT on LGA, the homogeneity hypothesis was rejected by the chi-square test (p<0.10, I^2^=70.2%), thus we selected the random-effects model and obtained the overall OR, and 95% CI was 2.08 (1.56, 2.78) (Figure 2).

Table 2 presents the results of subgroup analyses of the effects of gestational IGT on LGA. When stratified by geographic region, a positive association of gestational IGT and LGA was observed in the studies conducted in each region. We abstracted the ORs from the 14 studies, the analysis of the effects of gestational IGT on LGA yielded an overall adjusted OR of 2.36 (1.64, 3.37), but this apparent relationship was not observed in the analyses of the unadjusted ORs. The definitions of gestational IGT in these studies were different - some studies restricted the value of fasting plasma glucose (FPG) (4,5,14,15,23,24), the others just formulated the value of OGTT (16,17,18,19,20,21,22,25). When stratified by the unequal definition, the analysis of the effects of gestational IGT with restricted FPG value on LGA yielded an overall OR of 1.73 (1.01, 2.99). The definition of gestational IGT employed different forms of OGTT as well - for instance, some studies used the value of OGTT at 0, 60, 120, and 180 min (19,20,21,25), some used the value of 2-h 75-g OGTT (4,5,14,15,17,18,22,23,24), and one used the value of 1-h 50-g OGTT (16). When we stratified by the different forms of OGTT, a positive association of gestational IGT and LGA was obtained in the studies conducted in unequal definition.

Sensitivity analyses investigating the influence of the 14 studies individually on the overall risk estimate by excluding one study per iteration suggested that the overall risk estimates did not substantially change by any single study. The analysis of the effects of gestational IGT on LGA was with a range from a low of OR 1.7 (95% CI 1.49, 1.95) to a high of OR 2.19 (95% CI 1.66, 2.9). The results did not change substantially after sensitivity analysis.

### Publication Bias

**Figure 3:**
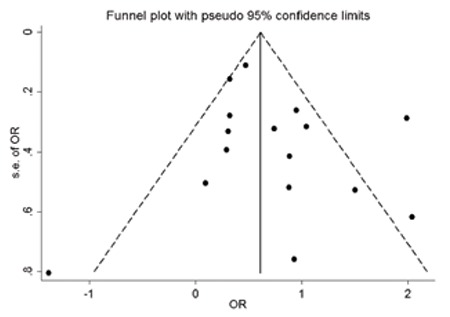
A funnel plot to visualize the publication bias of the 14 studies of this meta-analysis

In the funnel plot (Figure 3), we found that the scatters are substantially symmetric. There was no evidence of potential publication bias with the association of gestational IGT with LGA, as suggested by Egger’s test (p=0.314) and Begg’s test (p=0.499).

## DISCUSSION

The aim of our meta-analysis was to explore the association between gestational IGT and LGA. The results of a total of 14 epidemiologic studies of this meta-analysis showed that gestational IGT is an independent risk factor for neonatal LGA. Egger’s test and Begg’s test revealed no significant publication bias. The overall adjusted OR indicated that gestational IGT is an independent risk factor for neonatal LGA, and the overall combined OR of the effects of IGT with restricted FPG value on LGA also reflected this conclusion. When we stratified IGT by the different forms of OGTT, the consequences of analysis implied that the different forms of OGTT employed in the studies have no effect on our conclusion. When we excluded one study per iteration, the range of variation of the overall is also smaller suggesting that no one study can significantly alter the findings.

Gestational IGT is associated with postpartum metabolic dysfunction. Fetal growth in utero is a complex process and involves interactions among mother, placenta, and fetus. Mother’s and fetal endocrine statuses, genetic predisposition, and available substrates result in fetal growth, all of which also determine birth weight. However, since the placenta does not allow transfer of insulin to the fetus, a large fraction of maternal glucose is metabolized in the fetus, leading to fetal lipogenesis and excessive growth (26). Therefore, it is conceivable that gestational IGT may contribute to fetal growth and future high birth weight.

In earlier studies, we found that the achievement of glucose control in women with at least one abnormal OGTT value decreased adverse neonatal outcomes to near baseline levels (27,28,29). In current studies, when we compared women with gestational IGT to those with NGT, we found that gestational IGT was associated with adverse perinatal outcomes (such as preterm birth) as well as with LGA and macrosomia (16,18,22,30). We also found that in women without gestational diabetes, gestational IGT is an independent predictor of having a LGA infant (15).

It has previously been reported that LGA is linearly related to maternal plasma glucose levels (31,32). Physicians have always been concerned about GDM but were unaware of gestational IGT as a pre-GDM condition, and women with gestational IGT were being cared for in the same way as normal pregnant women. As the results of our meta-analysis have shown, gestational IGT is an independent risk factor for neonatal LGA. Today, it is known that the monitoring of blood glucose during pregnancy is important for the control of the frequency of neonatal LGA. At this point, it is worth mentioning that recently, clinical studies have demonstrated that early intervention can prevent the development of diabetes in women with IGT (33). However, intervention trials of gestational IGT have not yet been realized in clinical trials. Therefore, if treatment suggestions are to be introduced to women with gestational IGT, the effects of such suggestions on pregnancy outcomes will need to be evaluated, also taking social, cultural, economic, and clinical benefits into account.

In conclusion, the results of our meta-analysis have shown that maternal gestational IGT increased the risk of LGA infants and was an independent predictor for neonatal LGA. Additional studies are needed to evaluate whether the monitoring of blood glucose and control of blood sugar by means of lifestyle programs (e.g. physical activity, diet) are beneficial in reducing the risk of neonatal LGA. The use of potentially biased evidence was the principal limitation of this study since the definition of gestational IGT showed differences among the studies. However, the consequences of the subgroup analyses implied that the different definition of gestational IGT employed in the studies had no effect on our conclusion.

## Ethics

Ethics Committee Approval: Retrospective study, Informed Consent: Retrospective study.

Peer-review: External and Internal peer-reviewed.

## Figures and Tables

**Table 1:**
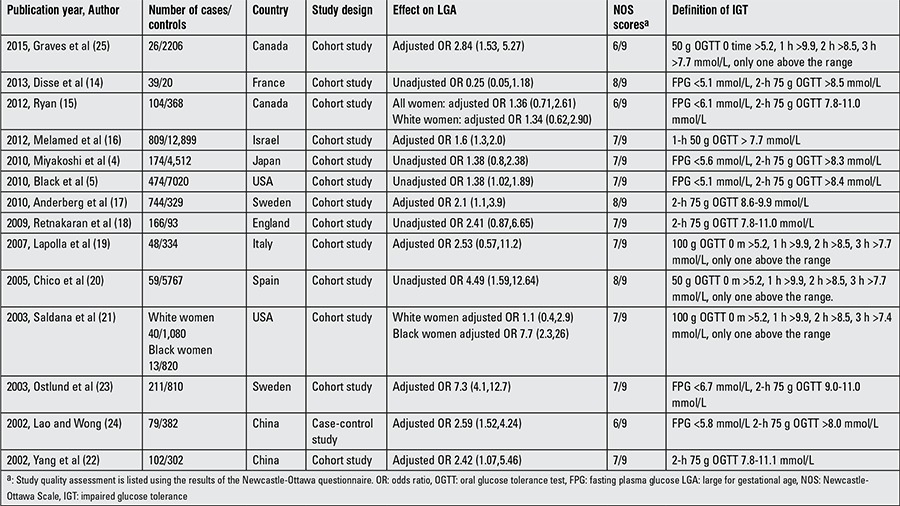
Characteristics of the studies included in the meta-analysis

**Table 2:**
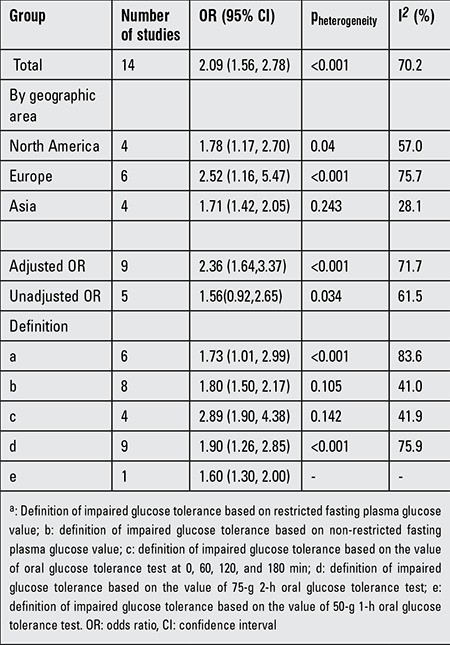
Sensitivity analysis of the effects of impaired glucose tolerance on large for gestational age
